# The Constancy of Colored After-Images

**DOI:** 10.3389/fnhum.2017.00229

**Published:** 2017-05-10

**Authors:** Semir Zeki, Samuel Cheadle, Joshua Pepper, Dimitris Mylonas

**Affiliations:** Laboratory of Neurobiology, University College LondonLondon, UK

**Keywords:** color, vision, color constancy, after-images

## Abstract

We undertook psychophysical experiments to determine whether the color of the after-image produced by viewing a colored patch which is part of a complex multi-colored scene depends on the wavelength-energy composition of the light reflected from that patch. Our results show that it does not. The after-image, just like the color itself, depends on the ratio of light of different wavebands reflected from it and its surrounds. Hence, traditional accounts of after-images as being the result of retinal adaptation or the perceptual result of physiological opponency, are inadequate. We propose instead that the color of after-images is generated after colors themselves are generated in the visual brain.

## Introduction

Color constancy refers to our ability to discount the wavelength-energy composition of the light in which a surface or object is viewed and assign a constant color to it. It has been discussed by many authorities, including Helmholtz (1867), Hering (1964), Rushton and Henry (1968) and Land (1974). By color constancy we do not mean that the color of a surface which is part of a complex scene maintains its exact hue, or shade of color, when viewed successively in lights of different wavelength composition. The hue will naturally change as the wavelength composition of the light reflected from it and its surrounds changes, becoming darker or lighter depending upon the predominance of one set of wavebands or another. Hence a better term would be a *constant color category*, and we use the term constant color to mean constant color categories.

In the work reported here, we investigated whether the color of the after-image of a patch, just like the color itself, depends on the ratio of the wavelength composition of the light coming from the patch and its surrounds. If so, this would have a significant bearing on understanding the extent to which the color of the after-image can be accounted for by adaptation or by physiological opponency; it should lead to a new view of how colored after-images are generated.

Viewing of a colored surface has a perceptual consequence, namely the subsequent perceptual appearance of a (negative) colored after-image that belongs to a family of colors which is approximately complementary to the one viewed (Burckhardt, 1866; Pridmore, 2008). This perceptual phenomenon was used by Hering (1964) in developing his opponent theory of vision, which Hurvich and Jameson (1957) established on a quantitative basis. Physiological opponency in the visual brain, from the retina onwards (Svaetichin, 1956; De Valois et al., 1966; Gouras, 1968; De Monasterio and Gouras, 1975; Derrington et al., 1984), acts to sharpen the spectral selectivity of chromatic cells, making them more responsive to narrower wavebands of light than the absorption spectra of the three receptors in the retina. Its discovery has played a significant role in accounting for perceptual color opponency in physiological terms.

The observed physiological wavelength opponency, where cells excited by long-wave light are inhibited by middle-wave light (or vice-versa), and cells excited by short-wave light are inhibited by long-wave plus middle-wave light, is irresistibly close to the documented perceptual color opponency charted quantitatively by Hurvich and Jameson (1957) and others (De Valois et al., 1966; Derrington et al., 1984). Yet the relationship, in terms of action spectra and peak wavelength selectively of cells on the one hand and the observed psychophysical color opponency on the other, is much too loose to enable us to account accurately for the perceptual system in terms of the physiological one, at least up to the primary visual cortex (area V1; Valberg, 2001; Gegenfurtner and Kiper, 2003).

This loose relationship makes it interesting to learn whether perceptual color opponency can be directly related to physiological opponency. We opted to study this within the context of color constancy, by asking whether the color of after-images is due to retinal adaptation or whether, like the color of the image itself, it is independent of the precise wavelength-energy composition of the light reflected from it but depends as well on the wavelength energy-composition of the light coming from its surrounds and the ratios between the two. If so, it should be, within wide limits, independent of the wavelength-energy composition of the light in which it is viewed (Land, 1974). A convenient approach was to extend Land’s classical Mondrian experiments (Land and McCann, 1971; Land, 1974, 1986). This approach had the advantage that it constituted a significant departure from the use of uniform monochromatic patches and surrounds employed by Anstis et al. (1978) to investigate whether the color of the after-image is dependent upon “simultaneous color contrast” or “induced colors”. It emphasized instead the colors of after-images when colored surfaces are viewed in more natural conditions, when they reflect light of all wavebands.

Our question can be formally summarized as follows: *Is the color of the after-image of a patch which is part of a multi-colored scene and illuminated by light of all wavebands due to adaptation to the dominant wavelength reflected from it or is it dependent on its color alone?* As an example, would viewing, say, a green surface that reflects more red light in a more natural context but is perceived as green (color constancy) result in a green after-image? This is what would be predicted from theories that aim to account for the color of the after-image through adaptation, retinal or otherwise, or through physiological wavelength opponency (Craik, 1940; Brindley, 1959; Rushton and Henry, 1968; Sakitt, 1976; Anstis et al., 1978; Virsu, 1978; Williams and MacLeod, 1979; Hofstoetter et al., 2004) or both. It could, on the other hand, result in a red after-image which is what would be predicted from viewing a green surface. If so, then the implication would be that it is not “chromatic adaptation”, whether retinal or otherwise, and not physiological wavelength opponent mechanisms either, that are the basis of the colored after-image. Rather, it would suggest that the color of the after-image is generated after the colors themselves are generated in the cortex. The work reported here therefore complements earlier physiological work undertaken with single cells in the cortex (Zeki, 1983a).

Our study is based on asking subjects to determine the color of the after-image produced by viewing colored patches which are parts of complex multi-colored scenes and reflect light of many wavebands. This is in contrast to previous studies in which the color of the after-image was produced by viewing colored stimuli isolated from all surrounds (Williams and MacLeod, 1979) or against neutral surrounds (Zaidi et al., 2012), and /or by looking at neutral (gray) spots against monochromatic surrounds (induction; Anstis et al., 1978). We by contrast made each viewed central patch of the multi-colored display reflect similar triplets of energies belonging to the long, middle and short-wavebands; under these conditions, each patch maintains its color category (color constancy).

## Materials and Methods

### Multicolored Displays

**Figure 1:**
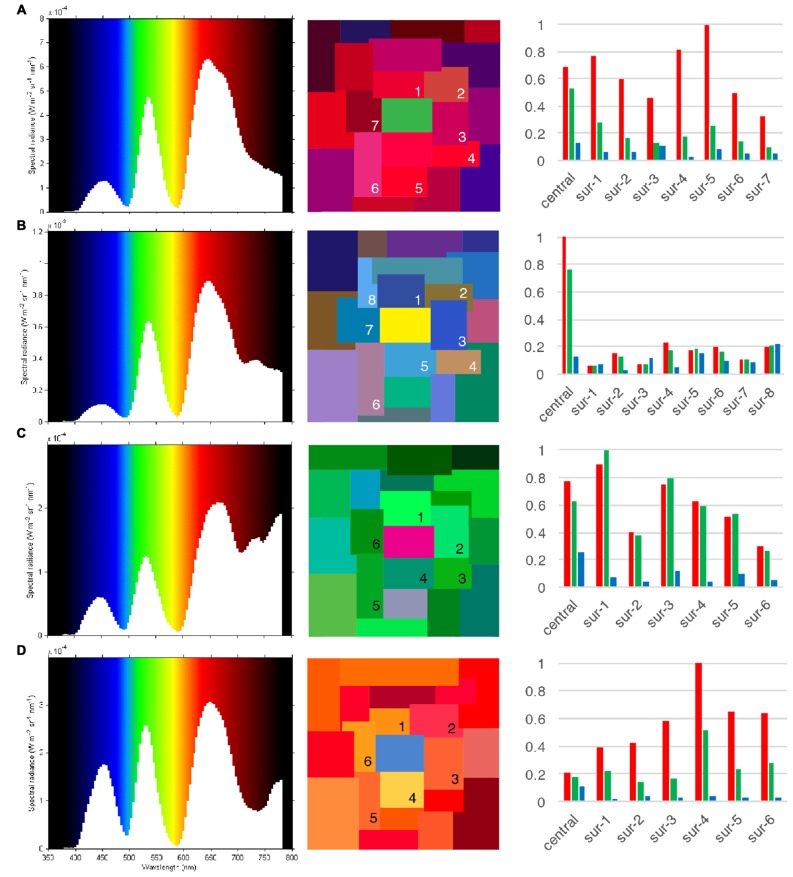
**The Mondrian displays used in this experiment and their characteristics**. Reflected energies from the four different Mondrian displays **(A–D)** are shown in left the column, color appearance in the center column, and LMS cone excitation ratios in the right column. The spectral power distributions of long (L), middle (M) and short (S) wave light reflected from each central patch (green, yellow, magenta, blue) are given in watts per steradian per metre square per wavelength (W Sr m^−2^ nm). LMS in 10° cone excitation ratios are given separately for the central patch, and for each patch immediately bordering the central patch, up to 10° from the central patch.

We used four Land color Mondrians to vary the wavelength composition of the light reflected from surfaces without changing their perceived color (see Figure 1). Each consisted of an arbitrary assembly of rectangular and square patches of different size (Max: 12.5° × 8°; Min: 5° × 4.5°) and color, arranged in such a way that there were no recognizable objects and none of the patches was surrounded by another patch of a single color. The patches were made of matte Color Aid papers, which reduce specular reflectance. The color of the surround patches, which extended more than 10° in all directions from the central patch (which subtended 8.25° × 6°), belonged to the family of colors complementary to the central patch (i.e., if the central patch was yellow, the surround patches were in various hues of purple and blue). The spectral power distributions are reported in watts per steradian per square meter (W Sr m^−2^ nm); they were obtained by measuring the light reflected from each viewed (central) patch with a PR-650 tele-spectroradiometer (see Figure 1). We also show the stimulus specifications for each Mondrian display in 10° relative cone fundamentals (Stockman and Sharpe, 2000), for the central patch and for each of the surrounding patches (see also Supplementary Material Table A1 in the Appendix for the numerical values of the cone excitation ratios). We use an extent of 10° from the central patch because experiments show that this is the critical spatial range beyond which the modulation of perceived color by its surround rapidly declines (Wachtler et al., 2001). Note that, in the A, B and D displays of Figure 1, the dominant waveband reflected from the central patch was also the dominant waveband reflected from the surrounds. In display C, the color of the green surround was the same as the color of the after-image that would be perceived after viewing the central (magenta) patch. This was done to ensure that the color of the after-image produced by viewing the central patch could not be accounted for by “color induction” produced by the surrounds (Anstis et al., 1978).

### Illumination of the Displays

Three 350W Kodak Carousel projectors equipped with rheostats were used to illuminate the Mondrians, as in the original Land Mondrian experiments; each was equipped with specially manufactured long, medium or short wave gelatin filters (Zeki, 1980). Projector 1 transmitted long wave light in the range of 592 nm to the end of the visible spectrum (peak transmittance greater than 660 nm); projector 2 transmitted middle wave light in the range 492–580 nm (peak 528 nm). The short-wave projector transmitted light in the range 386–493 nm (peak 432 nm) with a secondary peak at 700 nm. Each projector was equipped with a separate rheostat and shutter, thus enabling the intensity of light coming from each to be adjusted separately. The average luminance of the central patch, and of the surrounding patches extending 10° from the central patch in all directions was 14.30 cd/m^2^ for display A, 6.30 cd/m^2^ for display B, 4.19 cd/m^2^ for display C and 21.69 cd/m^2^ for display D.

### Color Selection Targets

To obtain as objective an account as possible of the colors of the after-images, subjects were asked to match the perceived colors of the after-images with color patches from the Munsell Book of Color (glossy collection—M40115). Ten approximately uniformly distributed patches of equal value (*V* = 6) and chroma (*C* = 8), and each subtending 2°, were displayed on an annulus at a constant eccentricity of 8° of visual angle. Observers matched the colors of the after-images to Munsell samples presented against a white background illuminated with two GrafiLite daylight simulators (CIE 1931 *x* = 0.327, *y* = 0.339, CCT = 5742); their task was to determine which patch was the closest match to that of the after-image they experienced.

### Observers

Six subjects (5 males; aged between 21 and 35 years) with normal or corrected to normal vision were recruited using the UCL online subject recruitment system (SONA).

### Adjustments of the Visual Displays

We adjusted the amount of long, middle and short wave light reflected from the central (target) patch so that under full illumination conditions (central patch + surrounding patches) it appeared its “normal” color (for example green), even when it was reflecting more light of wavebands that are of the complementary (opponent) family, for example red family in this instance (see Figure 1). In general, our aim was to make all four central patches reflect the same wavelength-energy composition of light from them while retaining their colors. We note that, if such a patch were to be viewed in the void mode (that is, isolated from the surrounds), it would appear white or a light gray while its after-image would be a neutral but darker gray.

### Testing

All four multi-colored displays were placed 2.4 m from the projectors and observers sat at a distance of 1.3 m from it. The projectors were adjusted appropriately for the particular Mondrian display and illuminated the entirety of the display (i.e., both the central patch and the surround). Any remaining light sources in the experimental room were eliminated.

Subjects fixated the central patch of each display for a period of 30 s and then reported verbally the color of the after-image by choosing one of the four predefined opponent color categories—blue, yellow, red or green. They next selected, from the 10 Munsell patches, the one that was the closest match to the color of the after-image which they perceived. The color selection targets were placed 60 cm from the observer on a rear desk. The task-lamps were turned on/off immediately after each response to allow the observers to adapt to the viewing conditions of each task while the experimenters were documenting either the verbal or the pointing response. Observers named the color of the afterimages while looking at a white board illuminated identically as in the stimulus presentation phase, and selected the closest Munsell match against a white background. The procedure was repeated three times to measure the reliability of after-image percepts, giving a total of 72 trials.

We emphasize that, in the natural viewing mode, when a patch reflected more light of a given wavelength, the surrounding patches were so chosen that they, too, reflected more light of the same wavelength. For example, if a green patch was made to reflect more long-wave light, the surrounds also reflected more long-wave light, even though each patch maintained its color (see Figure 1). This was to avoid any “induction” effects, as in the experiments of Anstis et al. (1978).

Thus, our experiments combine three approaches which have not been used before in color afterimage experiments: (a) the use of a multi-colored Mondrian scene; (b) making central patches of different color reflect similar wavelength-energy compositions of light; and (c) to overcome the fluctuating nature of afterimages, by using both a quick color naming task and a Munsell color matching task.

Before completing the main experiment, observers were tested on the Farnsworth-Munsell 100 Hue test and all were found to be within the normal range (Verriest et al., 1982).

## Results

Verbal reports of the colors of afterimages were grouped into three categories: (a) reports of opponent colored afterimages belonging to the family of colors that is complementary to the color of the target patch (Pridmore, 2008); (b) reports of non-opponent colored afterimages not belonging to the family of colors that is complementary to the test patch; examples are an after-image whose color was identical to the test patch, or colored afterimages that bore no clear opponent relationship to the test patch; and (c) no response reports were those in which subjects did not report seeing a colored after-image.

**Figure 2:**
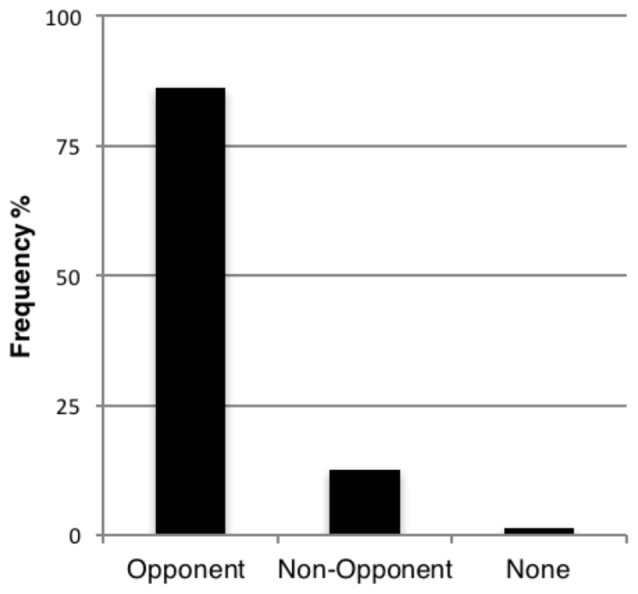
**Percentage of responses falling into the three main response categories, averaged across all subjects (*n* = 6)**.

The percentage of reports falling into each of the three categories, and averaged across six observers, was as follows (see Figure 2): the most frequently reported color for the after-image belonged to the opponent family (86.11% of trials), while colors not belonging to the opponent family were reported in 12.50% of trials. Observers reported perceiving no after-image in 1.39% of trials. As the primary aim of this experiment was to tabulate the color of the perceived after-image, we excluded all no response trials and considered only two types of response—opponent and non-opponent—for further analysis. A paired-sample *t*-test was conducted to compare the observations reporting opponent and non-opponent after-images. Overall, there was a significant difference in reporting opponent after-images (*M* = 2.58, SD = 0.93) and non-opponent ones (*M* = 0.38, SD = 0.92); *t*_(23)_ = 5.88, *p* < 0.0001 for all test displays.

**Figure 3:**
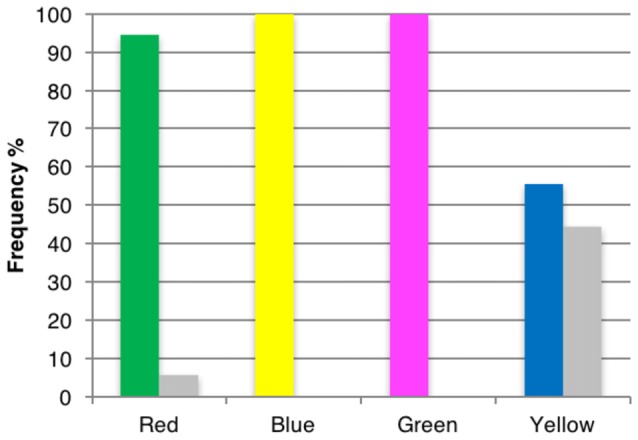
**Percentage of opponent and non-opponent colored after-images, for each of the four different display boards used**. The color of the bars correspond to the color of the central patches of displays A, B, C and D. Color names indicate the reported color of their After-Images. Gray columns correspond to non-opponent responses.

Figure 3 gives a breakdown of the results. Display A (green central patch) produced an opponent after-image in 94% of presentations, display B (yellow central patch) in 100%, display C (magenta central patch) in 100% and display D (blue central patch) in 56%. A paired-sample *t*-test between opponent (*M* = 2.83, SD = 0.41) and non-opponent (*M* = 0.17, SD = 0.41) after-images for display A revealed a significant difference: *t*_(5)_ = 8, *p* < 0.0001 using Bonferroni adjustments (*a* = 0.0125). For display B and C, we found no variance in the opponent and non-opponent responses—all observers reported an opponent after-image (i.e., 100%). For display D, we found no significant evidence that opponent (*M* = 1.67, SD = 1.51) after-images were reported more frequently than non-opponent (*M* = 1.33, SD = 1.51) ones; *t*_(5)_ = 0.27, *p* > 0.05 (see “Discussion” Section).

Hence, for all display types, opponent colors were perceived more often than “all non-opponent” colors, though the result was not significant for display D.

### Matching the Color of the Colored After-Images

**Figure 4:**
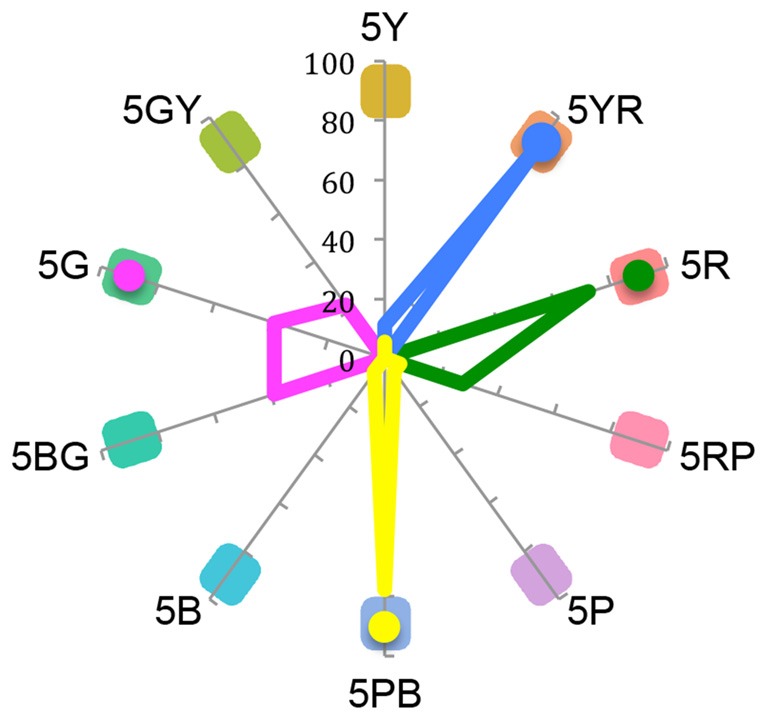
**Frequency distributions of color matching tasks are shown for all four Mondrian displays**. Chip colors and codes (Munsell specifications) are displayed along the polar dimension. Line colors correspond to the color appearance of the central patches (green, yellow, magenta, blue) and dots indicate the median.

The results of the color-matching task were analyzed in terms of frequency distributions (see Figure 4).

The most frequently reported color after viewing the green central patch (display A) was red (5R; 72%) while that for viewing the yellow central patch (display B) it was purple-blue (78%; 5 PB). For the magenta central patch (display C) subjects reported an after-image corresponding to blue-green and green (chips 5G and 5BG, 39% for each) and for the blue central patch (display D) they reported seeing yellow-red (5YR; 89%).

In summary, our results thus show that the color of the after-image produced by viewing a colored patch is independent of the precise wavelength-energy composition of the light coming from it, just as the color itself is.

## Discussion

We asked subjects to view central Mondrian patches which reflect the same wavelength-energy composition of light and yet maintain their color category (color constancy). We then asked them to report the color of the after-images produced by such viewings. The consistency with which the colored after-image belonged to the family of colors complementary to the viewed patch was 94% for red, 100% for blue, 100% for green and over 55% for yellow after-images. We were thus able to demonstrate that the color of afterimages is opponent to the perceived color. The color of the after-image therefore depends on the ratio of the wavelength-energy composition of the light reflected from the viewed patch and from its surrounds and is therefore independent of the precise wavelength-energy composition of the light reflected from the viewed patch alone. The uniformity of the color naming responses for the after-images follows the relative size of these terms (categories) in color spaces (Mylonas and MacDonald, 2016).

These results are generally consistent with previous reports on the colors of negative after-images (Burckhardt, 1866; Pridmore, 2008). There was nevertheless some variability which was more prominent for the blue central patch which, consistent with previous results (Stromeyer, 1969; Loomis, 1972), produced weaker and more ambiguous after-images. The reasons for this are not clear but may be related to the relatively lower spatial resolution of the visual system for short-wave (blue) light (Humanski and Wilson, 1992) and the smaller population of S than of L and M cones in the retina, especially in the fovea (Williams et al., 1981).

It is difficult to account for these results solely by assuming some kind of retinal von Kries local gain control, because the dominant waveband in the light reflected from the central patch was also the dominant waveband in the light coming from the surrounds, up to 10° in all directions, and because afterimages are opponent to perceived colors rather than wavelength, which von Kries mechanisms are assumed to operate on. The lateral extent of the horizontal cells in retina or of cells in V1 do not extend beyond 1–2° for central regions (Ts’o and Gilbert, 1988; Packer and Dacey, 2002) and so, to be effective, a von Kries type gain control would have to function over a considerable chain, more extensive than any that has so far been demonstrated. Hence, we agree with previous reports which have questioned the ability of retinal chromatic adaptation and the von Kries rule to fully account for color constancy (West and Brill, 1982; Worthey and Brill, 1986; Foster, 2011; Kulikowski et al., 2012; McCann and Rizzi, 2012).

To illustrate the inadequacy of cone contrast mechanisms to explain the formation of after-images, we can use as an example Display A (green central patch surrounded by reddish-purple and blue color patches), when the observers projected the colored after-image onto a white board, but the same applies for all viewing conditions. Let us assume that the von Kries rule applies and cones in the retina adapt independently or nearly independently to the central patch of the Mondrian when it is reflecting L, M and S wave light in the ratios of (*L* = 0.684, *M* = 0.530, *S* = 0.134) and to the surround patches (see Figures 1, 2 and Supplementary Material Table A1) in the averaged ratios of (*L* = 0.638, *M* = 0.177, *S* = 0.064). Note that, under these conditions, the L cones are stimulated by more light than M cones. After stimulus offset the mosaic of photoreceptors will generate a set of signals complementary to that generated by the stimulus. Where L, M and S cones had been highly stimulated by the Mondrian they are relatively less responsive to the white board and vice versa. Therefore, the signals sent by the retina would be the same as if looking in a reduction screen setting at the complementary color, greenish and green for proximal field and background area respectively. However, the fact that the color of the after-image for this display was significantly reported in both tasks as red and not green, as the cone adaptation hypothesis would predict, implies that colored after-images are constructed after the signals have left the receptors level in the retina and not until after colors themselves have been generated in the cortex.

The results cannot, as well, be accounted for by physiological wavelength opponency, of the kind demonstrated between retina and cortex (e.g., De Valois et al., 1966). The responses of wavelength opponent cells in V1, for example, have been found to correlate with wavelength composition of the light reaching the eye rather than with its color (Zeki, 1983a). The consequence is that such cells will respond to a surface of any color depending upon the excess, in the light reflected from the surface, of the wavelengths that excite or inhibit it.

The findings we present thus reinforce earlier conclusions which downgrade the importance of cone contrast (Von Kries rule) in the generation of color constancy and assign an increasingly important role to higher cortical mechanisms for the production of colored after-images (Rinner and Gegenfurtner, 2002; Murray et al., 2006). Collectively, all these results are consistent with a late stage model of after-image opponency which posits that colored after-images are generated only after the colors themselves are generated in the brain (Zeki, 1983a,b). This is not to say that physiological wavelength opponency mechanisms are not involved in endowing cells with responses that correlate with perceived hues (Zeki, 1983a; Conway et al., 2007; Brouwer and Heeger, 2009) but the nature of that involvement remains to be clarified.

The actual cortical site at which after-images are generated is less certain. It is likely that V4 plays a critical role. V4 is implicated in color vision (Zeki, 1980; Wade et al., 2008) in both monkey (Zeki, 1983a; Wild et al., 1985; Brewer et al., 2002; Conway and Tsao, 2006) and human brains (Zeki et al., 1991; McKeefry and Zeki, 1997; Wade et al., 2008; Goddard et al., 2011; Liebe et al., 2011; Lofar-Sousa et al., 2016) and the responses of cells in it can be selective for hues (Zeki, 1980; Conway et al., 2007; Brouwer and Heeger, 2013). As well, an imaging study of activity in cortical areas which correlate with the perception of colored after-images pinpointed V4 (Sakai et al., 1995). It is of course likely that V4 does not act in isolation but in cooperation with areas V1 and V2, both of which contain sub-compartments of cells which are wavelength selective or opponent and which are reciprocally connected with V4 (Shipp and Zeki, 1995). Although most cells in both V1 and V2 are perhaps more adequately described as wavelength selective, since their responses correlate with the wavelength composition of light as opposed to perceived color (Zeki, 1983a; Moutoussis and Zeki, 2002), weak surround effects, which may mediate color interactions, have been described in V1 (Wachtler et al., 2003).

### Color and Wavelength

Past studies of color vision have been heavily dominated by the use of uniform monochromatically illuminated patches, either in isolation or against monochromatically illuminated backgrounds. Although this has yielded a great deal of cardinal information, it nevertheless has restricted the study of color and of colored after-images to conditions which are not usually encountered in daily life. In more natural viewing conditions, the color of a surface or object is determined by reflection of light of all wavebands from it and from its surrounds, there being a crucial difference in the wavelength-energy composition of the light reflected from the two. It is this difference that ultimately determines color constancy (Land, 1974). Adopting this more natural stimulation condition, we have shown here that, just as perceived colors are constructed by spatial ratio taking operations, so are the perceived colors of the after-image.

We thus make a distinction between adaptation to color when produced by monochromatic light in the usual standard reduction screen setting of a laboratory and when produced by viewing an object or surface that is part of a complex scene when both the surface and its surrounds reflect light of many wavebands. They both produce a colored after-image, but the nature of the colored after-image cannot be adequately studied, or explained, using monochromatic surrounds alone, as done in the studies of Anstis et al. (1978). In particular, it is not possible to tell whether the colored after-image depends on the wavelength-energy composition of the light reflected from a surface or not. It is important to make the distinction between adaptation to color and adaptation to wavelength. We argue that the former is perceptually potent, while the latter, assuming it to exist, is not. This also brings into focus the inadequacy of terms such as “retinal chromatic adaptation”, however much they have come into usage. It perpetuates an historical confusion between wavelength and color (see also Zeki, 1983a,b). While lights of specific wavelength are perceived as having specific colors, the reason for this is traceable to the same laws that operate to generate color, whether attributable to single wavebands of light or to complex configurations where a surface reflects light of all wavebands (Land and McCann, 1971; Land, 1974). Hence study of after-images using monochromatic light has given an inadequate account of the role of physiological opponency in particular, and color opponency in general, in the generation of color by the cerebral cortex.

In summary, the results of the present study, in conjunction with the evidence described above, indicates that colored afterimages are the result of a late stage mechanism. The traditionally accepted view of photoreceptor adaptation cannot account for our results and nor can the physiological wavelength opponency observed in the visual pathways from retina to area V2. Our evidence is in favor of a cortical basis for the generation of colored afterimages, one that occurs after colors are generated.

## Ethics Statement

This study was carried out in accordance with the recommendations of the UCL Research Ethics Committee. All subjects gave written informed consent. No vulnerable populations were involved.

## Author Contributions

Experiment conceived by SZ. All co-authors took part in the experiments. Statistical analyses were done by SC. Colorimetry by DM. Article written by SZ and DM.

## Conflict of Interest Statement

The authors declare that the research was conducted in the absence of any commercial or financial relationships that could be construed as a potential conflict of interest. The reviewer DP and handling Editor declared their shared affiliation, and the handling Editor states that the process nevertheless met the standards of a fair and objective review.
