# Supplementary material for: The Constancy of Colored After-Images
Source: Front Hum Neurosci. 2017 May 10;11:229. doi: 10.3389/fnhum.2017.00229 (PMC5423953; doi:10.3389/fnhum.2017.00229)
Supplement: Supplementary file 1 [file Data_Sheet_1.docx]

Appendix A.

|  | Target A (green) | | | Target B (yellow) | | | Target C (magenta) | | | Target D (blue) | | |
| --- | --- | --- | --- | --- | --- | --- | --- | --- | --- | --- | --- | --- |
|  | L | M | S | L | M | S | L | M | S | L | M | S |
| central | 0.684 | 0.530 | 0.134 | 1.000 | 0.766 | 0.125 | 0.770 | 0.627 | 0.250 | 0.211 | 0.182 | 0.104 |
| surround1 | 0.772 | 0.274 | 0.063 | 0.058 | 0.056 | 0.071 | 0.898 | 1.000 | 0.076 | 0.388 | 0.225 | 0.015 |
| surround2 | 0.599 | 0.160 | 0.063 | 0.154 | 0.123 | 0.026 | 0.399 | 0.381 | 0.035 | 0.430 | 0.141 | 0.041 |
| surround3 | 0.459 | 0.123 | 0.110 | 0.077 | 0.076 | 0.115 | 0.756 | 0.794 | 0.118 | 0.588 | 0.167 | 0.026 |
| surround4 | 0.817 | 0.179 | 0.030 | 0.231 | 0.178 | 0.043 | 0.626 | 0.590 | 0.042 | 1.000 | 0.512 | 0.039 |
| surround5 | 1.000 | 0.255 | 0.087 | 0.178 | 0.187 | 0.153 | 0.512 | 0.533 | 0.095 | 0.651 | 0.231 | 0.033 |
| surround6 | 0.495 | 0.146 | 0.050 | 0.196 | 0.162 | 0.093 | 0.296 | 0.268 | 0.049 | 0.645 | 0.281 | 0.029 |
| surround7 | 0.325 | 0.100 | 0.045 | 0.104 | 0.110 | 0.086 | NA | NA | NA | NA | NA | NA |
| surround8 | NA | NA | NA | 0.201 | 0.211 | 0.215 | NA | NA | NA | NA | NA | NA |

Table A1. Summary of long (L), middle (M) and short (S) wave light reflected from the four different Mondrian in 10^o^ cone excitation ratios. LMS ratios are given separately for the central patch, and for each patch immediately bordering the central patch, up to 10^o^ from the central patch.
